# Longitudinal Detection and Persistence of Minority Drug-Resistant Populations and Their Effect on Salvage Therapy

**DOI:** 10.1371/journal.pone.0135941

**Published:** 2015-09-11

**Authors:** Masako Nishizawa, Masakazu Matsuda, Junko Hattori, Teiichiro Shiino, Tetsuro Matano, Walid Heneine, Jeffrey A. Johnson, Wataru Sugiura

**Affiliations:** 1 AIDS Research Center, National Institute of Infectious Diseases, Tokyo, Japan; 2 Clinical Research Center, National Hospital Organization Nagoya Medical Center, Nagoya, Japan; 3 Infectious Disease Surveillance Center, National Institute of Infectious Diseases, Tokyo, Japan; 4 Division of HIV/AIDS Prevention, Centers for Disease Control and Prevention, Atlanta, Georgia, United States of America; 5 Department of AIDS Research, Nagoya University Graduate School of Medicine, Nagoya, Japan; University of Pittsburgh, UNITED STATES

## Abstract

**Background:**

Drug-resistant HIV are more prevalent and persist longer than previously demonstrated by bulk sequencing due to the ability to detect low-frequency variants. To clarify a clinical benefit to monitoring minority-level drug resistance populations as a guide to select active drugs for salvage therapy, we retrospectively analyzed the dynamics of low-frequency drug-resistant population in antiretroviral (ARV)-exposed drug resistant individuals.

**Materials and Methods:**

Six HIV-infected individuals treated with ARV for more than five years were analyzed. These individuals had difficulty in controlling viremia, and treatment regimens were switched multiple times guided by standard drug resistance testing using bulk sequencing. To detect minority variant populations with drug resistance, we used a highly sensitive allele-specific PCR (AS-PCR) with detection thresholds of 0.3–2%. According to ARV used in these individuals, we focused on the following seven reverse transcriptase inhibitor-resistant mutations: M41L, K65R, K70R, K103N, Y181C, M184V, and T215F/Y. Results of AS-PCR were compared with bulk sequencing data for concordance and presence of additional mutations. To clarify the genetic relationship between low-frequency and high-frequency populations, AS-PCR amplicon sequences were compared with bulk sequences in phylogenetic analysis.

**Results:**

The use of AS-PCR enabled detection of the drug-resistant mutations, M41L, K103N, Y181C, M184V and T215Y, present as low-frequency populations in five of the six individuals. These drug resistant variants persisted for several years without ARV pressure. Phylogenetic analysis indicated that pre-existing K103N and T215I variants had close genetic relationships with high-frequency K103N and T215I observed during treatment.

**Discussion and Conclusion:**

Our results demonstrate the long-term persistence of drug-resistant viruses in the absence of drug pressure. The rapid virologic failures with pre-existing mutant viruses detectable by AS-PCR highlight the clinical importance of low-frequency drug-resistant viruses. Thus, our results highlight the usefulness of AS-PCR and support its expanded evaluation in ART clinical management.

## Introduction

Combination antiretroviral therapy (ART) has reduced AIDS-associated morbidity and mortality in patients infected with HIV [1,2]. However, despite the development of highly effective antiretrovirals (ARVs), therapy does not eradicate HIV infections [3] and life-long treatment is necessary. In the course of long-term treatment, emergence of drug-resistant HIV which reduces the effectiveness of ART can threaten virologic suppression of HIV [4,5]. Current treatment guidelines including those from the US Department of Health and Human Services (DHHS) recommend HIV drug-resistance testing prior to ART initiation to assist in the selection of effective drugs and to avoid treatment failure by pre-existing drug resistance [6–9]. Bulk sequencing of the viral RNA in the plasma from an HIV-infected patient is a standard method to detect drug resistant HIV and has been widely used in a clinical setting. However the method is relatively insensitive, and does not detect low frequency variants that comprise less than 20% of the viral population in individuals [5,10–12]. More sensitive methods, such as point mutation assay (allele-specific PCR, AS-PCR) [13–15], oligonucleotide ligation assay (OLA) [16] and deep sequencing [17–21] have been developed to analyze minority populations carrying drug resistance mutations. Because deep sequencing technique tends to be expensive and more labor-intensive, particularly for resource-limited settings, low-cost AS-PCR is a useful tool to analyze minority population of drug resistance variants, especially for specific mutations of interest. The detection limit of AS-PCR method that we developed was previously calculated to be 0.3% as highest and 2% lowest sensitivities, depending on the target sites, for analysis of NRTI- and NNRTI-resistant mutations [14]. Several reports have suggested that pre-existing minority variants persist at low frequencies and associated with increased risk of virologic failure [10,11,13–19,21,22]. Several reports described that minority-level NNRTI drug resistance persisted for several months to over a year in individuals given single-dose NVP [23–25]. Low-frequency K103N was detected by highly-sensitive method for 6 to 12 month after the mutation was no longer detectable by bulk sequencing. Although many reports suggested that there is no impact of minority-level drug resistance mutations on ARVs [17,25–28], there is still much to learn of the significance of combination mutations in different ART regimens. Therefore, the ability to detect minor population of drug resistance mutations below 20% would improve identification of infections involving drug-resistant viruses and better inform decisions on their clinical consequences and the selection of active ARVs. Epidemiological surveillance of transmitted drug-resistant HIV in newly diagnosed treatment-naïve patients in Japan showed that the prevalence of resistance transmission rose from 5.9% to 8.3% (2003–2008) by bulk sequencing [29]. However, drug resistant mutations were detected in 26.8% of patients screened by AS-PCR [30]. This result indicated the underestimation of transmitted drug resistance mutations, and these undetectable minority-level drug resistance mutations by bulk sequencing may affect first-line ART. In this study, we used AS-PCR to analyze expression dynamics of eight drug resistance mutations (M41L, K65R, K70R, K103N, Y181C, M184V and T215F/Y) during the clinical course of ARV-treated individuals with documented virologic failures and drug-resistant HIV-1. Phylogenetic analysis was also used to better understand the possible impact of these low-frequency drug-resistant variants on the clinical course in these patients.

## Materials and Methods

### Patients and their profiles

In this study, six individuals were enrolled. All were Japanese men who have sex with men (MSM) and were infected with subtype B. Thus, the patients were from the major demographic of HIV-infected people in Japan (Table 1) [29]. All cases were on-ART at the time this of their enrollment to the study (S2–S7 Tables). A total of 180 plasma samples were collected from the six patients from November 1996 to July 2007. All individuals had been taking ARVs for more than 5 years, during which the treatment regimens were switched multiple times because of the difficulties in controlling viremia (S2–S7 Tables). Multiple samples were obtained from each case, 30 sampling points with three- to four-month intervals. As all cases were in virological failure, and most of the VL at the time of collection above 1000 copies/ml. However, we also attempted to amplify lower VL samples as well. There are only eighteen sampling points with longer intervals, six months to a year, in case 6 (S7 Table). Treatment regimens and period of treatments are summarized in Table 1.

**Table 1:** Demographics and clinical data.

Patient ID	Gender	Subtype	Viral load(copies/mL)			CD4 count			Observation period(month)	1st regimen	ARVs included in the regimens		Resistance mutations by bulk-sequencing	
			min	max	median	min	max	median			NRTI	NNRTI	NRTI	NNRTI
1	M	B	100	130700	10300	151	227	180	65	d4T/3TC	AZT, d4T, 3TC, ABC, TDF	NVP, EFV	M41L, M184V, T215Y	K103N, Y181C
2	M	B	100	1200000	45850	184	319	199	103	d4T	AZT, ddI, ddC, d4T, 3TC ABC	NVP	M41L, K70R, M184V, T215Y	Y181C
3	M	B	2000	60100	10450	42.6	262	109	85	AZT,3TC, IDV	AZT, 3TC, TDF	EFV	M41L, K70R, M184V	K103N
4	M	B	900	358800	33900	80	1999	265	104	ddI	d4T, ddI, 3TC, TDF	EFV	M41L, M184V, T215F	K103N
5	M	B	100	849500	26300	36	434	80	101	AZT	AZT, ddI, ddC, d4T, 3TC, ABC, TDF	EFV	M41L, M184V, T215Y	K103N, Y181C
6	M	B	2000	443500	36500	126	569	371	102	AZT	AZT, ddI, ddC, d4T, 3TC, ABC, TDF	NVP, EFV	M41L, M184V, T215Y	K103N, Y181C

### Ethics statement

Specimens were anonymous and residual diagnostic material from subjects who provided written consent for HIV testing. The Ethical Committee for Biomedical Science of the NIID determined that this testing did not involve identifiable human subjects and has approved the study. All testing was performed at the National Institute of Infectious Diseases (NIID), AIDS Research Center in Tokyo, Japan.

### RNA extraction and virus template amplification

HIV RNA copy number of each sample was measured using the Amplitaq Monitor v 1.5 (Roche Diagnostics, Indianapolis, IN). The detection limit of viral load was set to <50 copies / mL plasma in this study. CD4-positive T cell count data was obtained from clinical information supplied from hospitals where the individuals were treated. HIV RNA was extracted from patient plasma as described previously [30]. Briefly, HIV RNA was extracted from 200 μL of patient plasma and eluted into 100 μL of elution buffer. Extracted RNA samples were stored in -80°C until use. The HIV PR-RT region was amplified by one-step RT-PCR (TAKARA One Step RNA PCR kit) with a forward primer (DRPRO5: AGA CAG GYT AAT TTT TTA GGG A) and a reverse primer (DRRT34: GCT ATT AAG TCT TTT GAT GGG TCA TA). RT-PCR amplification conditions were 55°C for 40 minutes for the RT reaction followed by 40 cycles of 95°C for 10 seconds, 52°C for 5 seconds, and 72°C for 90 seconds. In the individual that the amplification of RT-PCR did not generate sufficient template, nested-PCR was performed using a forward primer (PROFWD1F: CAG ATC ACT CTT TGG CAA CGA CC) and a reverse primer (GEN4R: ATC CCT GGG TAA ATC TGA CTT GC) [30]. Nested-PCR amplification conditions were 94°C for 1 minute and 30 cycles of 94°C for 10 seconds, 55°C for 4 seconds and 74°C for 15 seconds.

### Conventional in-house drug resistance genotyping

All samples were analyzed by bulk sequencing employing our in-house protocol as reported elsewhere [30]. In brief, protease-RT regions (1144bp) were RT-PCR amplified followed by nested-PCR. Sequence reaction was performed by BigDye Terminator v3.1 Cycle Sequencing Kit, and analyzed by ABI-3100 auto sequencer. Drug resistance mutations were defined according to the mutation list proposed by Stanford HIV Drug Resistance Database [31].

### Highly sensitive AS-PCR

To detect low-frequency subpopulations with drug resistance mutations, we used highly sensitive AS-PCR validated for subtype B HIV as described previously [14]. Briefly, mutation-specific primers were designed for seven reverse transcriptase inhibitor resistance mutations, M41L, K65R, K70R, K103N, Y181C, M184V, and T215F/Y (S1 Table). According to the specificity of the primers, T215F primer cross reacted with T215L/I/ L/V, and T215Y primer with T215D/N /Y (S1 Table). The HIV-1 total copy primers, ComFWD and ComREV, that span nucleotides 258–420 in RT were used with the common probes, Com1P and Com2P (S1 Table) [14]. For multiple mutation screening, several resistance mutation-specific reactions can be performed simultaneously. The cycle number at which the fluorescence emission exceeds the background fluorescence threshold is the threshold cycle (CT) and is the unit of measure for comparing the differences in amplification signals (ΔCT) between the total copy and mutation-specific reactions. All samples were tested in duplicate with the means of the total copy and mutation-specific CTs used for the determination of the ΔCT. Each ΔCT cutoff value for interpreting the presence of drug resistance mutations was determined previously [14] and were between 8.5 to 10.5 amplification cycles, for validated assay cut-offs ranging from 0.3% to 2.0% mutant, depending on the assay. AS-PCR for detection of each drug-resistant mutation was performed following the conditions previously described [30].

### Assessing mutation associations in mutation-specific amplicons

To evaluate the genetic relationship between minority-level K103N variants detected by AS-PCR and those detected by bulk sequencing, amplicons from AS-PCR reactions that yielded positive results were sequenced and phylogenetic trees were constructed. To increase the genetic sequence information for phylogenetic analyses, we extended the K103N-specific amplicon to the 181-184.REV reverse primer, which allowed amplification of RT codons 103 to 220 (336 bp) (S1 Table). The T215I-positive amplicons were similarly analyzed with amplicons spanning codon 215 to 101. To avoid possible bias by K103N and T215I mutation in the phylogenetic analysis, K103N and T215I codon positions were removed from the sequences before analysis. The sequences were aligned by the Clustal W program with a set of reference sequences recommended by the Los Alamos sequence database (http://www.hiv.lanl.gov/content/index). Maximum likelihood phylogeny was inferred by MEGA6 [32] based on the General Time Reversible model with gamma distribution (5 categories) and invariable sites [33].

## Results

### Drug resistance mutants present at low frequency were detected by AS-PCR in 5 of 6 individuals

**Fig 1:**
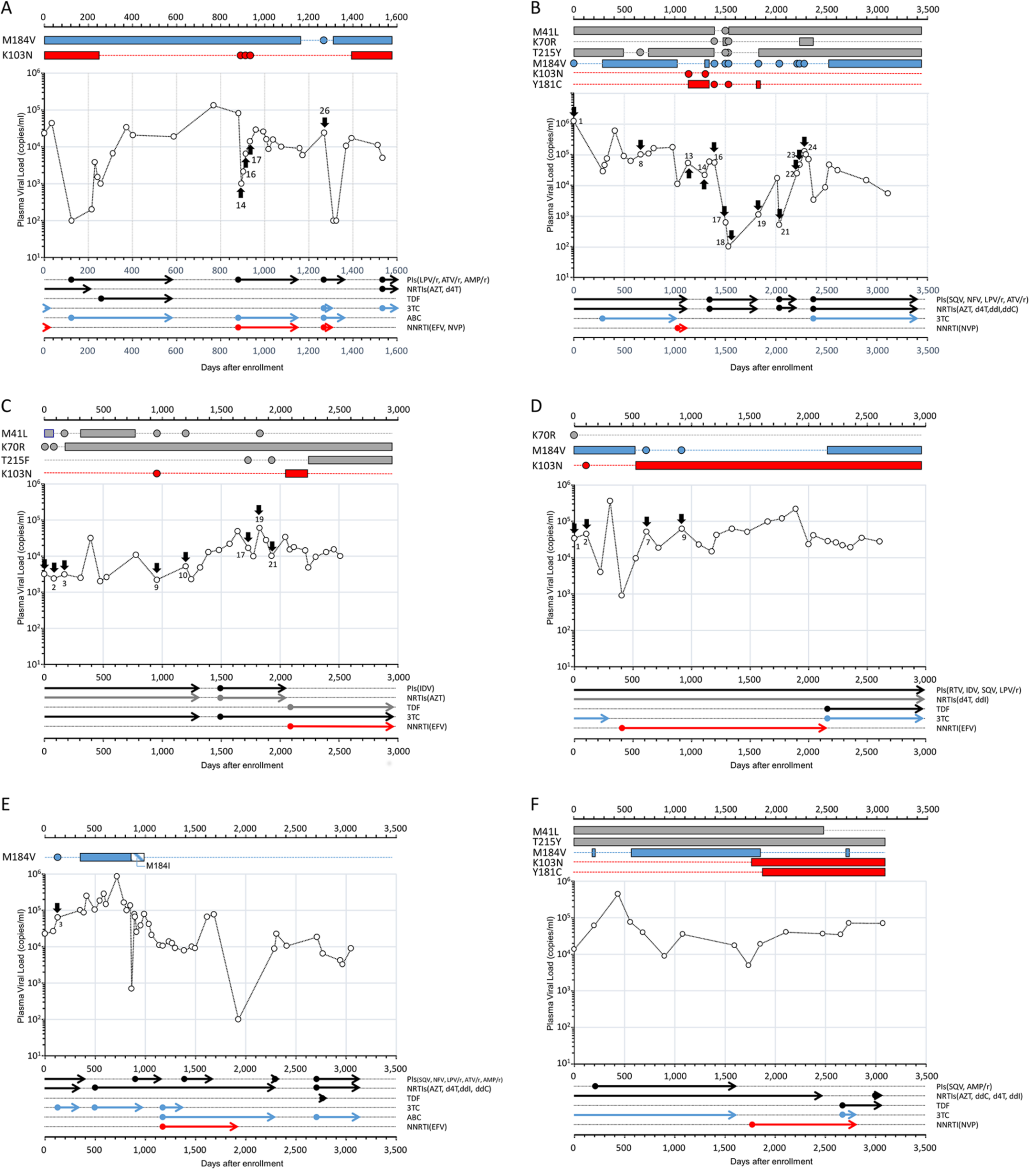
Chronologies of ART regimens, drug resistance mutations detected by bulk sequencing and AS-PCR, and change in viral loads in enrolled individuals. The chronological tables of (i) drug resistance mutations, (ii) viral load and (iii) ART regimens are depicted for each individual (A-F). (i) Resistance mutations detected: Bulk sequencing and AS-PCR results are shown in solid bars and closed circles, respectively. Their colors are matched with corresponding antiretrovirals shown in the ART regimen part of the table. (ii) Viral load: The arrows in each graph indicate the point that minor-drug resistance mutations were detected. The numbers indicated the ID of samples (S2–S6 Tables). (iii) ART regimens: Solid circles indicate the starting point of each regimen and the arrows indicate the period of each regimen.

Drug resistance mutations were detected by bulk sequencing in all 6 individuals who continued to experience insufficient viral suppression despite receiving salvage therapies in attempts to control viremia (Fig 1A–1F), (S2–S7 Tables). AS-PCR detected drug resistance mutations in 5 of 6 individuals in time points that had previously undetectable mutations by bulk sequencing as shown in S2–S7 Tables.

### Drug resistance mutations existed as minority populations for several years before being detected by bulk sequencing

K70R, K103N, M184V and/or T215I were detected at minority levels in 4 of 6 individuals several years prior to detection by bulk sequencing (Individual 2, 3, 4 and 5). In individual 2, K70R was detected as minority population by AS-PCR prior to bulk sequencing and continued to persist for >1000 days. (Fig 1B and S3 Table). In individual 3, K70R, K103N and T215I were detected as minority populations by AS-PCR, though not continuously, at least once before these mutations became detectable by bulk sequencing (Fig 1C and S4 Table). K103N and T215I were detected by bulk sequencing soon after the initiation of regimen which included EFV, followed by slight decrease of plasma VL (Fig 1C). Evidence of K103N and T215I minority-level populations were identified in specimens collected 30 and 16 months, respectively, prior to the time point at which resistance was detected by direct sequencing. Similarly, in individual 4, K103N was detected as a minority subpopulation 14 months prior to becoming detectable by bulk sequencing. (Fig 1D). In individual 5, M184V was detected by AS-PCR nearly 6 months prior to detection by bulk sequencing (Fig 1E). In individual 6, drug resistance mutations were detected as major population throughout the observation period, and no minority drug resistance mutations were detected (Fig 1F).

### Drug resistance populations persisted as minority populations after withdrawal of antiretroviral drug selective pressures

Persistence of drug-resistance variants after withdrawal of ARV drug-selective pressures were observed by AS-PCR in 4 of 6 individuals (Individual 1, 2, 3, and 4). In individual 1, K103N was selected during nevirapine treatment and continuously detected for 8 months. The mutation became undetectable by bulk sequencing 6 months after the treatment was switched to a protease inhibitor containing regimen. However, a K103N-possessing variant persisted at low frequency for nearly 2 years without nevirapine administration and was re-selected when EFV was used (Fig 1A). Similar patterns of minority resistance persistence were observed in other individuals; such as Y181C and M184V. In individual 2, K103N was detected twice by AS-PCR but never became major population (Fig 1B). In individual 4, M184V was detected sporadically as a low-frequency variant after 3TC was withdrawn from their regimen, suggesting a 3TC-selected minority population had persisted around the threshold of AS-PCR detection limit. M41L was also found as minority population in individual 3 by AS-PCR. It persisted for more than 2 years after it became undetectable by bulk sequencing (Fig 1C).

### Phylogenetic relatedness of majority and minority-level variants with K103N- or T215I

**Fig 2:**
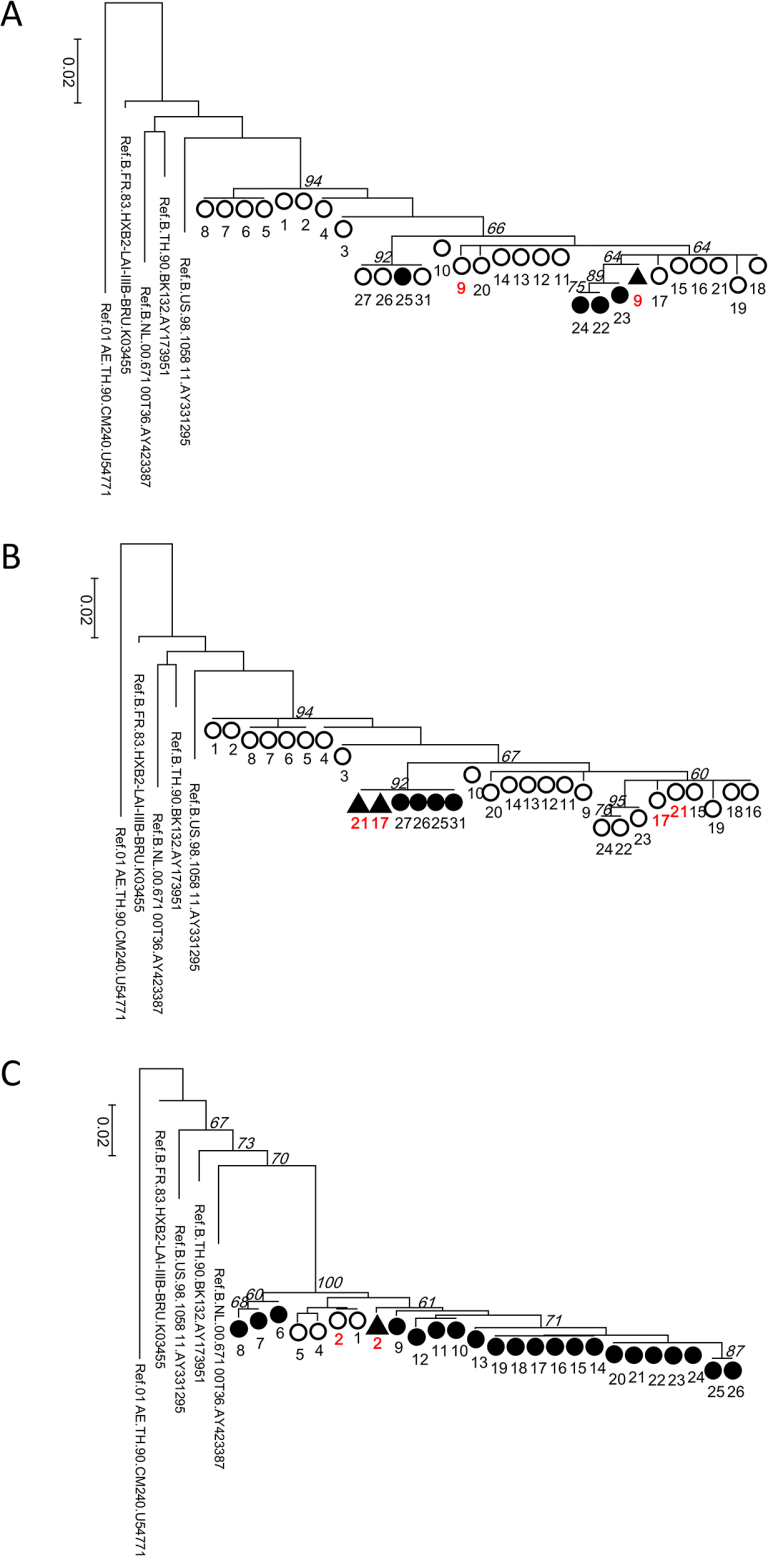
Phylogenetic analyses of AS-PCR amplicon and bulk sequences. Genetic relationships between pre-existing minority drug resistance populations and majority-level drug resistant populations that subsequently arose were analyzed by Maximum Likelihood phylogeny inferred by MEGA6. Numbers next to each symbol indicates the collecting point IDs of each patient (S4 Table and S5 Table). Italic numbers at the tree-nodes indicate bootstrap values of the taxa analyzed. (A) Analysis of K103N populations in Individual 3: Solid and open circles indicate K103N positive and negative bulk sequence results, respectively. A solid triangle indicates K103N-positive amplicon at ID 9. ID 9 samples are highlighted with red. (B) Analysis of T215I populations in Individual 3: Solid and open circles indicate T215I positive and negative bulk sequence results, respectively. Two solid triangles indicate T215I-positive AS-PCR amplicon from ID 17 and 21 samples. ID17 and 21 are highlighted with red. (C) Analysis of K103N populations in Individual 4: Solid and open circles indicate K103N positive and negative bulk sequence results, respectively. A solid triangle indicates sequence data of K103N-positive amplicon derived from ID 2 sample. Red letters; ID 2 samples are highlighted with red.

To clarify whether minority-level drug-resistant variants retrospectively detected by AS-PCR were the origins of majority resistant variants possessing the same mutations that later emerged, phylogenetic relatedness was assessed. In the phylogenetic tree for individual 3, the ID9 (day 957) AS-PCR amplicon (K103N positive) clustered with the K103N bulk sequence at ID 22–24 (days 2046–2117), suggesting that the K103N minority population at ID 9 had high genetic identity to the K103N-positive population that subsequently arose at ID22-24 (Fig 2A, S4 Table). A similar finding was observed for T215I positive populations in Individual 3. Two T215I-positive AS-PCR sequences on ID 17(day 1731) and 21 (day 1930) were clustered with ID 25–27 and 31(days 2215–2303, 2552), suggesting their close genetic relationships (Fig 2B, S4 Table). In Individual 4, the sequence of the K103N-positive amplicon detected at ID 2 (day 102) was within the cluster of K103N-positive sequences collected at later period ID 9–26 (days >917) (Fig 2C, S5 Table). The results of the three phylogenetic analyses suggested that pre-existing minority drug resistances may affect subsequent treatment outcome, supporting clinical importance of detecting minority-levels of drug-resistant variants prior to treatment changes.

## Discussion

To better understand the clinical significance of persistent minority-level drug resistance, we used the highly sensitive AS-PCR method to screen plasma specimens from 6 HIV-1-infected individuals treated with ARV for more than five years. All of these individuals had difficulty in controlling viremia because of low adherence and acquisition of multiple drug resistant mutations and, as a result, treatment regimens were switched multiple times. By comparing the drug resistance mutations detected by bulk sequencing and by our AS-PCR method, we were able to associate minority-level drug-resistant variants with poor treatment outcomes in two individuals. In these two individuals, persons 3 and 4, we confirmed that minority–level drug-resistant variants enriched by earlier ARV regimens had persisted for extended periods and subsequently became the major populations. Phylogenetic analysis showed that K103N, which was detected as minority by AS-PCR before treatment with EFV, became the major population after the regimen included EFV was initiated. This result demonstrated that minor-K103N may have been a factor in treatment failure after switching to the EFV-containing regimen. One limitation in this study is the lack of the plasma samples collected before treatment initiation, thus the origin of these minority-level resistant variants was unknown. These viral subpopulations may have originated as transmitted drug resistance strains [34–36], however, given the long treatment history, was likely selected from prior therapy.

As seen with K103N in individual 1, and M184V in individuals 2 and 4, once the resistant strains were selected, they persisted as minority populations even after their corresponding ARVs were withdrawn, to re-emerge soon after their selective antiretroviral drugs were readministered. These observations follow previous reports by others that some drug resistance mutations disappeared quickly after treatment interruptions or upon release of drug selective pressures, but rebounded when the therapies were restarted [37–40]. Notably, the detection of minority levels of resistant variants was not continuous but sporadic for mutations, such as K103N in individual 1 where minority K103N was detected only 3 times among 21 samples (14.3%) between day 311 and 1369, suggesting that the minority K103N population existed around the threshold level of AS-PCR which is 1% of the total population. Similar findings were observed for individual 2, in which M184V was detected in 8 of 12 time points (day 1393–2373) and T215Y in 1 of 3 (day 565–793). Likewise for individual 4, M184V was detected at 2 of 13 time points (day 616–2042) (S5 Table). Although it may be difficult to distinguish, detection of minority variants could be either a result of mutation decay from recent therapy or long persistence of low-frequency variants. Therefore, the detection of minority M184V in individual 4 and minor-M41L in individual 3 might reflect decay kinetics rather than persistence. It would not be possible to conclude from our study that minority-level drug-resistant variants had impacts in all treatment-failure cases. However, in some cases the phylogenetic analysis has clarified the outgrowth of minority-level K103N, suggesting that pre-existing K103N variants may have affected subsequent treatment failure. Thus, though the clinical significance of AS-PCR has been reported [10,11,13–19,21,22–24], fluctuations in minority variant expression may cause missed diagnosis of low-frequency resistance.

Although the clinical impact of minority population of NRTI-resistant mutations on ART outcome is still under discussion [12,26], our previous report and findings in this study demonstrated the importance of detecting minority-level drug-resistant variants. Here we present evidence for M184V and K103N, which are key resistance mutations and frequently observed in both treated [41,42] and newly diagnosed individuals [43–47]. Our highly sensitive AS-PCR may provide further insight into the clinical relevance of drug resistance mutations producing data at least as informative yet much less costly than Sanger sequencing, of particular benefit to low-middle income countries (LMIC) where ART usage has been expanding. Next generation sequencing (NGS) analysis, a powerful new technology that enables deeper evaluation of HIV genetic diversity [19,46,48,49], will add to a better understanding of the still conflicting clinical relevance of drug-resistant minority variants. However, the utility of AS-PCR for detecting key mutations to particular regimens of interest is not lost. Earlier studies comparing AS-PCR and NGS have found that, for mutations to specific ARVs of interest, the results from the two methods were similar. The benefit is that the AS-PCR results were obtained much quicker and at a fraction of the cost and labor of NGS, an important consideration for clinical management in LMIC.

## Supporting Information

S1 Table Oligonucleotide sequence Proportion (RTI mutations). (XLSX)

S2 Table Regimens, drug resistance mutations of NRTI/NNRTI by bulk-sequencing and AS-PCR in individual 1. (XLSX)

S3 Table Regimens, drug resistance mutations of NRTI/NNRTI by bulk-sequencing and AS-PCR in individual 2. (XLSX)

S4 Table Regimens, drug resistance mutations of NRTI/NNRTI by bulk-sequencing and AS-PCR in individual 3. (XLSX)

S5 Table Regimens, drug resistance mutations of NRTI/NNRTI by bulk-sequencing and AS-PCR in individual. (XLSX)

S6 Table Regimens, drug resistance mutations of NRTI/NNRTI by bulk-sequencing and AS-PCR in individual 5. (XLSX)

S7 Table Regimens, drug resistance mutations of NRTI/NNRTI by bulk-sequencing and AS-PCR in individual 6. (XLSX)
